# Proteomic Profiling of an Exosome-Enriched Extracellular Vesicle Fraction and Structural Characterization of SMPDL3A in the Carcinogenic Liver Fluke *Clonorchis sinensis*

**DOI:** 10.3390/ijms27020682

**Published:** 2026-01-09

**Authors:** Seon-Hee Kim, Dongki Yang, Young-An Bae

**Affiliations:** 1 Research & Business Development Foundation, The State University of New York, Korea, Incheon 21985, Republic of Korea; sunnykim14@gmail.com; 2 Department of Physiology and Lee Gil Ya Cancer and Diabetes Institute, Gachon University College of Medicine, Incheon 21999, Republic of Korea; 3 Department of Microbiology and Lee Gil Ya Cancer and Diabetes Institute, Gachon University College of Medicine, Incheon 21999, Republic of Korea

**Keywords:** trematode parasite, *Clonorchis sinensis*, exosomes, excretory-secretory products, proteomics, SMPDL3A, sphingolipid metabolism, host–parasite interaction

## Abstract

Exosomes are important mediators of host–parasite communication and contain diverse molecules that may support the survival of *Clonorchis sinensis* in the biliary tract. To explore their biochemical properties, exosomes isolated from excretory–secretory products of Korean *C. sinensis* isolates were characterized through integrated morphological, proteomic, and gene ontology analyses. The vesicles exhibited typical exosomal size ranges and marker profiles, and their protein components were enriched for cytoskeletal, metabolic, and vesicle-trafficking components relevant to epithelial signaling and immune modulation. Among these proteins, sphingomyelin phosphodiesterase acid-like 3A (SMPDL3A) was examined in detail to obtain molecular evidence suggesting its role in sphingolipid metabolism in the parasite. The *C. sinensis* SMPDL3A (Cs_SMPDL3A) shared the overall structure and core catalytic residues with mammalian homologs, SMPDL3A and sphingomyelin phosphodiesterase 1 (SMPD1), a finding consistent with the possibility that Cs_SMPDL3A may retain authentic sphingomyelinase activity. Although lacking the saponin B domain of SMPD1, Cs_SMPDL3A carries a C-terminal transmembrane segment that may facilitate sphingomyelin access by positioning the enzyme on lipid bilayers. Collectively, these findings suggest that Cs_SMPDL3A participates in host sphingomyelin turnover, potentially generating ceramide for uptake by SMPD1-lacking *C. sinensis* or contributing to ceramide-associated immune responses in the biliary tract, offering new insight into lipid-centered host–parasite interactions during clonorchiasis.

## 1. Introduction

*Clonorchis sinensis* (phylum Platyhelminthes; class Trematoda) is a food-borne liver fluke endemic to East Asia, particularly Korea, China, and Vietnam, where millions of people remain at risk of infection through the consumption of raw or undercooked freshwater fish [1,2]. After ingestion, the metacercariae excyst in the duodenum and migrate into the intrahepatic bile ducts, where they develop into adult worms capable of surviving for decades [1,3]. Chronic infections can lead to cholangitis, bile duct hyperplasia, periductal fibrosis, hepatic cirrhosis, and ultimately cholangiocarcinoma [1,2,4]. Given the strong epidemiological and mechanistic association with the biliary cancer, *C. sinensis* has been designated a Group I biological carcinogen together with its closest phylogenetic neighbor *Opisthorchis viverrini* [5]. Despite its considerable public health significance, the molecular basis of parasite-host interactions that underlie biliary pathology and carcinogenesis remains incompletely understood.

Over the years, investigations have focused on the identification of bioactive molecules generated by *C. sinensis* that support long-term parasite survival in the biliary tracts and/or represent promising candidates for diagnostic antigens and therapeutic vaccines. These parasite-derived factors encompass diverse biochemical classes, including enzymes, structural proteins, metabolic regulators, protease inhibitors, and glycan-rich macromolecules, each carrying distinct roles in host–parasite communication (reviewed in [6]). Proteins involved in nutrient acquisition and metabolic compensation help the parasite overcome its restricted anabolic capacities, a characteristic broadly observed in parasitic flatworms due to reduced biosynthetic pathways [7,8,9,10,11]. In parallel, immunomodulatory proteins influence macrophage activation, lymphocyte differentiation, and the balance between pro- and anti-inflammatory responses, shaping both early inflammatory reactions and chronic tolerance [6,12]. Other secreted components that act on cholangiocytes, hepatic stellate cells, or epithelial stress pathways promote tissue remodeling and periductal fibrosis, creating microenvironments favorable for parasite persistence ([6] and references cited therein). Although many of these molecules have been profiled across developmental stages of *C. sinensis*, the specific pathways through which they collectively shape pathogenic outcomes remain poorly defined, underscoring the need for deeper mechanistic investigation.

Parasitic helminths secrete a spectrum of soluble or extracellular vesicle (EV)-associated macromolecules, collectively termed excretory-secretory products (ESP), which mediate host–parasite communication, enable nutrient acquisition, and modulate host immunity [13]. Among these secretions, molecules enclosed in or bound to EVs, particularly exosomes, have attracted growing attention as specialized and highly effective mediators of the host interaction [14,15,16]. Whereas soluble ESP proteins exert broad effects on host tissues, EVs provide a unique functional advantage by encapsulating proteins, lipids, and RNAs within a lipid bilayer that allows protected transport and direct delivery of cargo into target cells via membrane fusion or receptor-mediated uptake [17,18]. Recent findings show that *C. sinensis* EVs activate IL-6 and TNF-α secretion in biliary epithelial cells through a TLR9-mediated ERK pathway [19], and promote aberrant proliferation, migration, and epithelial–mesenchymal transition (EMT) in cholangiocarcinoma cells through NF-κB/EMT signaling [20]. In a related study, Pan et al. [20] reported the first proteomic profile of EV proteins from Chinese *C. sinensis* isolates, together with a brief gene ontology (GO)-based categorization of the identified proteins [20]. Studies in related trematodes such as *O. viverrini* and *Fasciola gigantica* have shown that their exosome-like vesicles contain conserved cytoskeletal, metabolic, and vesicle-trafficking proteins that influence epithelial signaling, immune activation, and tissue remodeling [21,22], reflecting conserved molecular machineries of the host–parasite communication in these hepatobiliary trematode parasites.

Lipid metabolism represents a particularly constrained aspect of host-dependent anabolism in parasitic flatworms, since the de novo synthesis of fatty acids and sphingolipids necessitate high energetic costs. To date, parasite-secreted proteins involved in fatty acid acquisition, including fatty acid-binding proteins and lipases, have been relatively well characterized, whereas molecular components associated with sphingolipid assimilation remain poorly understood in these organisms [10,11]. Given the conserved structural and signaling roles of sphingolipids in eukaryotic membranes [11], it is plausible that parasites deploy specific molecules to support their own sphingolipid homeostasis. In this context, ESP and/or EV represent biologically relevant platforms through which sphingolipid-related enzymes may be selectively enriched and functionally delivered at the host–parasite interface. In this study, an exosome-enriched EV fraction was obtained from Korean *C. sinensis* isolates and subjected to proteomic characterization. Among the identified proteins, a putative acid sphingomyelin phosphodiesterase-like enzyme, sphingomyelin phosphodiesterase acid-like 3A (SMPDL3A), was further examined to explore its potential involvement in parasite-associated sphingolipid metabolism. These findings provide new insight into the molecular architecture and putative functions of *C. sinensis* exosomes, offering a refined perspective on how the parasite establishes, maintains, and exacerbates clonorchiasis, while also yielding molecular clues relevant to sphingolipid remodeling in parasitic flatworms.

## 2. Results

### 2.1. C. sinensis Exosomes

**Figure 1:**
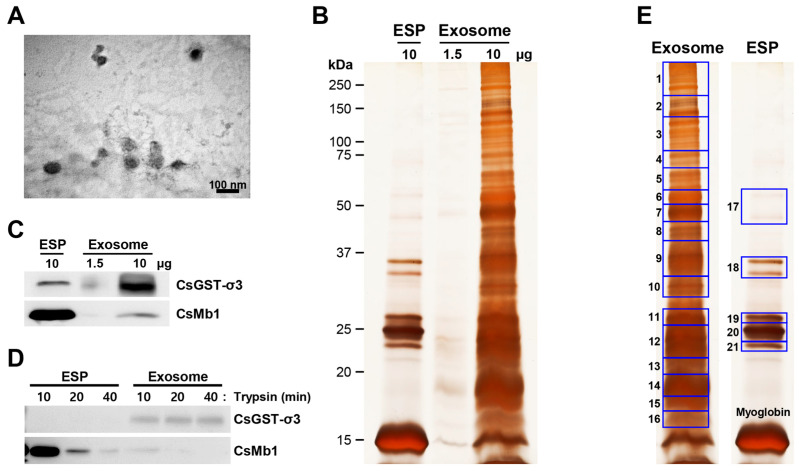
Morphological and biochemical characterization of *Clonorchis sinensis* exosomes. (**A**) Transmission electron microscopy image of exosomes ranging from 40 to 90 nm in diameter (100,000× magnification). (**B**) SDS-PAGE analysis of whole ESP (10 μg) and exosomal fractions (1.5 and 10 μg). (**C**) Western blot analysis of ESP and exosomes probed with anti-CsMb1 and anti-CsGST-σ3 antibodies. (**D**) Western blot analysis of trypsin-treated ESP and exosomes (2 μg each) probed with the same antibodies. (**E**) SDS-PAGE gel sections excised for LC-MS/MS protein identification. Protein bands in panels (**B**,**E**) were visualized using silver staining on 10–15% polyacrylamide gels. Note that several highly abundant ESP protein bands clustered around approximately 15, 25, and 35 kDa dominate the ESP lane under the standardized silver-staining development conditions used to optimize EV protein visualization.

Exosomes contained in the ESP of *C. sinensis* adults were collected using the exosome isolation reagent and identified through a transmission electron microscope (TEM). The TEM image showed multiple spheroids with heterogenous diameters ranging from 40 to 90 nm (Figure 1A). Whole ESP displayed a protein profile dominated by several highly abundant bands clustered around approximately 15, 25, and 35 kDa (Figure 1B), consistent with findings from a previous study [23]. Under the standardized silver-staining development conditions used to optimize EV protein resolution, lower-abundance ESP components were comparatively less visible. In contrast, these specific bands were markedly reduced in the exosomal fraction, whereas several other bands became relatively more apparent, consistent with selective enrichment in this fraction. Myoglobin (CsMb1; AAM18464) and sigma-class glutathione transferase (CsGST-σ3; ABC72085), which were the most and second most abundant proteins in *Clonorchis* ESP, respectively [23,24], were readily detected among these protein bands by Western blot analysis, although their relative abundances were reversed in the exosomal fraction (Figure 1C). Notably, the myoglobin-specific signal was undetectable in trypsin-treated ESP and exosome fraction, whereas the GST-specific band remained unaffected (Figure 1D). This result demonstrated that myoglobin is secreted into ESP in free form and was not completely removed during exosome isolation, likely due to its high abundance in the starting material. Based on the observed banding patterns, the SDS-PAGE gel lane, excluding the myoglobin band, was divided into 21 sections (16 from the exosomal fraction lane and five from the whole ESP lane; Figure 1E). Gel pieces from each section were excised and processed by in-gel trypsin digestion and liquid chromatography-tandem mass spectrometry (LC-MS/MS) for proteomic identification.

### 2.2. Clonorchis Proteins in Exosomes

MASCOT analysis of peptide masses obtained from the 21 gel sections identified 232 and 226 protein matches in two *C. sinensis* proteomes, independently established by genome projects based on Korean (Proteome-K) and Chinese (Proteome-C) isolates, respectively (Supplementary Tables S1 and S2). Many proteins were redundantly detected across multiple gel sections, whereas 28 proteins in each proteome were identified exclusively in the ESP lane (Supplementary Table S3). After consolidating redundant identifications and restricting downstream analyses to proteins detected in the exosomal fraction, a total of 97 and 89 unique proteins were identified in the peptide mass fingerprinting (PMF) analysis of Proteome-K and Proteome-C, respectively (Supplementary Tables S4 and S5). Highly represented proteins, inferred from the number of matched peptides in the PMF analysis, included structural or membrane-associated components such as basement membrane-specific heparan sulfate proteoglycan core protein, a GPI-anchored surface glycoprotein, TRPM8 channel-associated factor 2, and CD63; metabolic enzymes such as propionyl-CoA carboxylase, glutamate dehydrogenase, and fructose-bisphosphate aldolase; and the stress-related protein ferritin heavy chain (Supplementary Tables S1 and S2). These patterns are consistent with the enrichment of metabolic, structural, and stress-response proteins commonly reported in helminth exosomal proteomes.

Most proteins were shared, but minor discrepancies were observed between the two datasets. To account for these discrepancies and reconcile corresponding proteins across datasets, orthogroups were established by comparing the retained proteins of Proteome-K and Proteome-C. Reciprocal BLASTp searches (ver. 2.17.0+; *E*-value = 0; identity > 90% within the aligned region; coverage > 50%) were used to identify corresponding proteins between proteomes (Supplementary Tables S4 and S5). Matches with <90% identity were also accepted if reduced values were due to large alignment gaps, likely arising from errors in exon-intron annotation. The resulting protein pairs were then cross-validated against those identified by PMF, and proteins absent from only one PMF-based list were reintroduced if their absence was attributable to mispredicted gene structures. In total, 104 and 101 orthogroups were established as the K- and C-set exosomal proteins, respectively (Supplementary Table S6), and these refined sets were subsequently subjected to GO analysis.

### 2.3. Functional Categories of Clonorchis Exosomal Proteins

**Figure 2:**
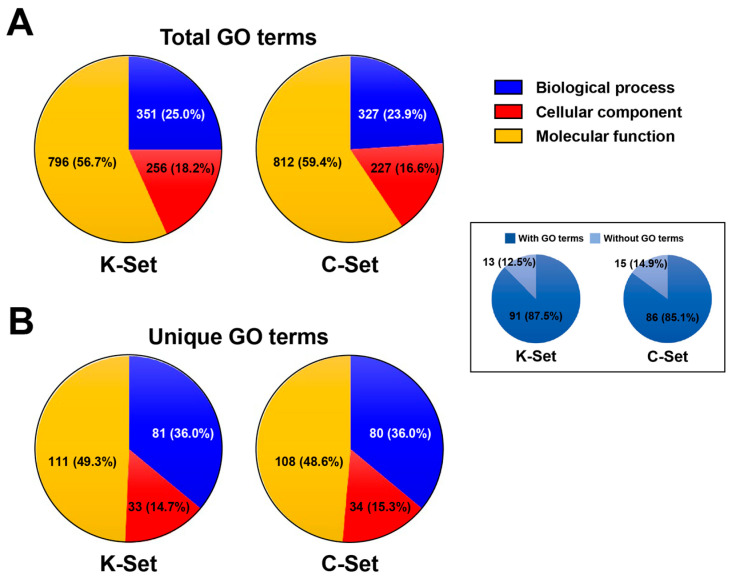
Gene Ontology (GO) term distributions of *Clonorchis sinensis* exosomal proteins. Total (**A**) and unique (**B**) GO terms assigned to exosomal proteins are shown across the biological process (blue), cellular component (red), and molecular function (orange) categories. The GO terms were determined from the K- and C-set proteomes, which were inferred from the Korean and Chinese isolate genomes, respectively. Pie charts in box indicate the proportions of orthogroups containing at least one GO term (dark blue) versus those lacking assignable domains (light blue).

InterProScan assigned a total of 640 and 617 GO terms to 91 (87.5%) and 86 (85.1%) proteins of the K- and C-set exosomal proteins, respectively, with hierarchical redundancy, while the remaining 13 (12.5%) and 15 (14.9%) proteins were not assignable (boxed pie charts in Figure 2). Through propagation of the domain-based raw terms using the full ontology (go-basic) and slim mapping, these counts expanded to 1403 and 1366 terms, respectively. The resulting terms were classified into the three major ontology categories: biological process (BP; 25.0% in K-set and 23.9% in C-set), cellular component (CC; 18.7% in K-set and 16.6% in C-set), and molecular function (MF; 56.7% in K-set and 59.5% in C-set) (Figure 2A). When condensed to unique, non-redundant assignments, the numbers decreased to 225 and 222 for the K- and C-sets, respectively, with broadly consistent distributions across the three categories (Figure 2B).

**Figure 3:**
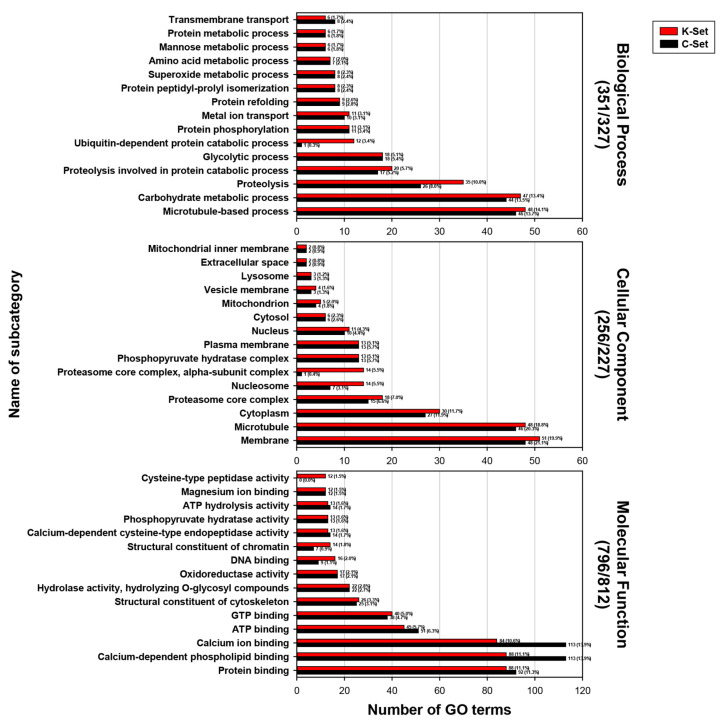
Enriched GO subcategories in *Clonorchis sinensis* exosomal proteomes. Terminal GO terms from the K- and C-set proteomes were grouped into the 15 most abundant subcategories within the biological process, cellular component, and molecular function categories, respectively. Numbers in parentheses indicate the total number of GO terms assigned to each proteome (K-set/C-set). Histogram bars show the absolute counts and percentages for each subcategory (red, K-set; black, C-set).

For a more detailed functional profile of exosomal proteins, GO terms within each major category were further resolved into terminal subcategories. In the K-set exosomal proteins, the most abundant GO terms under BP were associated with microtubule-based processes and the catalytic degradation of macromolecules, including carbohydrates and proteins (upper histograms in Figure 3). Within CC and MF, the dominant annotations corresponded membrane and cytoplasm components (middle histograms), and to functions related to protein and nucleotide bindings (lower histograms), respectively. Annotations related to calcium metabolism were also prominent in MF. A comparable subcategory profile by abundance was observed in C-set proteins (black bars in Figure 3), although several subcategory terms, such as ubiquitin-dependent protein catabolic process in BP; proteasome core complex, alpha-subunit complex and nucleosome in CC; and cysteine-type peptidase activity, structural constituent of chromatin, and DNA binding in MF, showed notable differences. Given the close correspondence of GO-assigned orthogroups between the K- and C-sets (Supplementary Table S6), these discrepancies were most likely attributable to mis-predicted exon-intron architectures in the parallel *C. sinensis* genome projects, despite the overall comparability of their quality indicated by the main category distribution patterns.

### 2.4. Sphingomyelin Phosphodiesterase Acid-like 3A and Its Homologs

**Figure 4:**
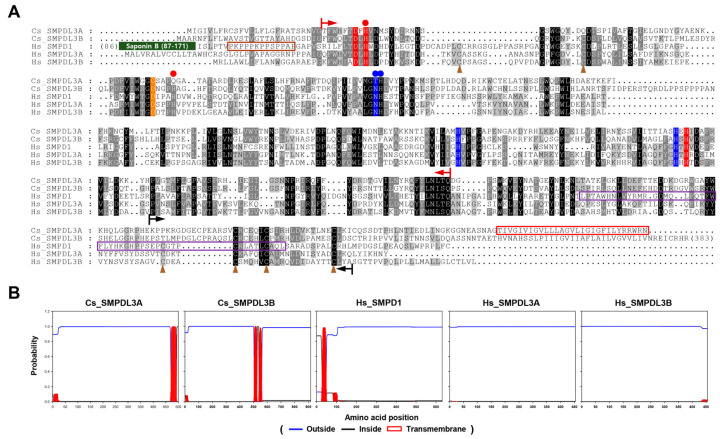
Structural comparison of Cs_SMPDL3A and homologous proteins. (**A**) Conserved amino acid residues and functional domains in Cs_SMPDL3A, Cs_SMPDL3B, Hs_SMPDL3A, Hs_SMPDL3B, and Hs_SMPD1. Metallo-dependent phosphatase domains are indicated by rust red arrows, ASM_C domains by black arrows, the Pro-rich linker by a brown box, the SMPD1 C-terminal domain by a purple box, and the TM_EGFR-like domain by a red box. Zinc-coordinating residues (Zn1, red; Zn2, blue) and the Zn-bridging residue (orange) are highlighted. Residues interacting with substrate phosphoryl O3 and O2 atoms are marked by red and blue circles, respectively. Disulfide-bond-forming cysteines are indicated by brown arrowheads. (**B**) DeepTMHMM predictions of signal peptides and transmembrane segments.

Of the diverse proteins identified in the *Clonorchis* exosomes, SMPDL3A (orthogroup 048 in Supplementary Table S6), also known as acid sphingomyelinase (ASM)-like protein 3A, was selected for further characterization to gain molecular insight into sphingolipid metabolism in this liver fluke. The protein, designated *C. sinensis*_SMPDL3A (Cs_SMPDL3A), was composed of 498 amino acids (aa), and contained two conserved domains, metallo-dependent phosphatase (IPR029052; aa 27–346) and ASM carboxy (C)-terminal region (ASMase_C, IPR045473; aa 297–441) domains (Figure 4A). In addition, a strong hydrophobic segment corresponding to a transmembrane_epidermal growth factor receptor-like (TM_EGFR-like) domain (CDD cd12087; aa 470–498), which is implicated in both membrane anchorage and dimerization [25,26], was detected at the C-terminus (Figure 4B).

BLASTp searches using the Cs_SMPDL3A sequence retrieved one and three homologous proteins from *C. sinensis* and human proteomes, respectively. The *Clonorchis* (Cs_SMPDL3B, KAG5454866.1/GAA35962.2) and human (Hs_SMPDL3A, Hs_SMPDL3B, and sphingomyelin phosphodiesterase 1 [Hs_SMPD1]) homologs shared the metallo-dependent phosphatase and ASMase-C domains. Hs_SMPD1, which is synonymous with ASM, contained two additional functional features in its N-terminal region, saponin B-type domain (Sap_B, IPR008139) and Pro-rich linker [27]. None of these homologs possessed the TM-EGFR domain at their C-termini (Figure 4B).

Within the metallo-dependent phosphatase domain, aa residues coordinating two zinc ions (highlighted in red for Zn1 and blue for Zn2) and bridging them (orange) [27,28] were clearly identified at the corresponding positions, although the bridging Glu was substituted with Gly in Cs_SMPDL3A. In addition, residues interacting with two oxygen atoms (O2, blue circles and O3, red circles) of the substrates’ phosphoryl group, as well as Cys residues forming conformation-stabilizing disulfide bonds (brown arrowheads) [27], were relatively well conserved among these *Clonorchis* and human homologs (Figure 4A).

### 2.5. Tertiary Structures of SMPDL3A Homologs

**Figure 5:**
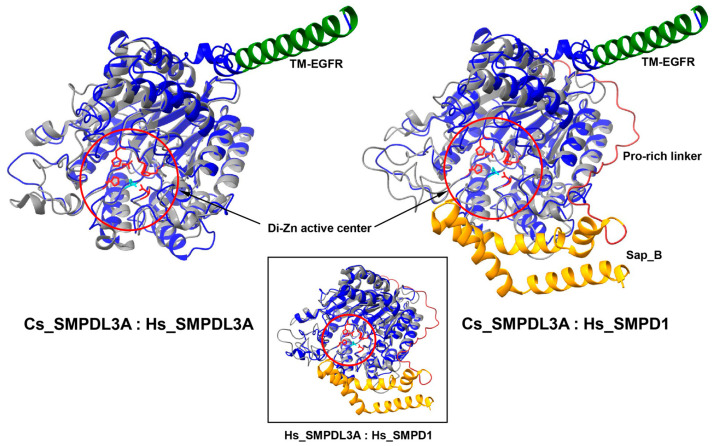
Tertiary structures of Cs_SMPDL3A and homologous proteins. AlphaFold-predicted Cs_SMPDL3A structure (blue) was aligned with Hs_SMPDL3A and Hs_SMPD1 structures (grey). Conserved di-zinc catalytic centers are highlighted with red circles, with zinc-coordinating residues shown in red and the Zn-bridging residue in cyan. The TM-EGFR and Sap_B domains conserved in Cs_SMPDL3A and Hs_SMPD1 are indicated in green and orange, respectively. The inset shows an additional structural alignment between human SMPDL3A (grey) and SMPD1 (blue).

Tertiary structures of Cs_SMPDL3A and Cs_SMPDL3B were predicted using AlphaFold (predicted template modeling score, 0.88 and 0.85, respectively). In these models, 90.8% and 86.4% of aa residues were located within the most favored regions of the Ramachandran maps, and the corresponding average geometry factors were 0.11 and 0.05, indicating good overall model quality (Supplementary Figure S1). The Cs_SMPDL3A structure was well aligned with those of Hs_SMPDL3A and Hs_SMPD1 with root mean square deviation of 0.909 Å over 286 and 1.008 Å over 247 pruned Cα pairs, respectively (Figure 5). Comparable structural topology was also observed between Cs_SMPDL3B and each of the human homologs (0.831 Å over 300 and 0.966 Å over 276 pruned pairs), as well as between the two human homologs (0.946 Å over 316 pruned pairs), reflecting a higher degree of structural similarity among these *Clonorchis* and human homologs. In these alignments, the di-zinc active center occupied a conserved niche with a similar surrounding conformation (highlighted in red circles in Figure 5), although the Sap_B domain (orange), which was connected to the central catalytic domain by the highly kink-prone Pro-rich linker (brown), was oriented near the catalytic entryway in Hs_SMPD1. The domain might create a hydrophobic interface with the active center that could accommodate sphingomyelin in a sandwich-like manner, as described previously [27]. Meanwhile, no structural counterpart of the C-terminal hydrophobic α-helix in Cs_SMPDL3A (TM_EGFR; green) was defined in the human proteins.

### 2.6. Evolutionary Features of Cs_SMPDL3A Homologs

**Figure 6:**
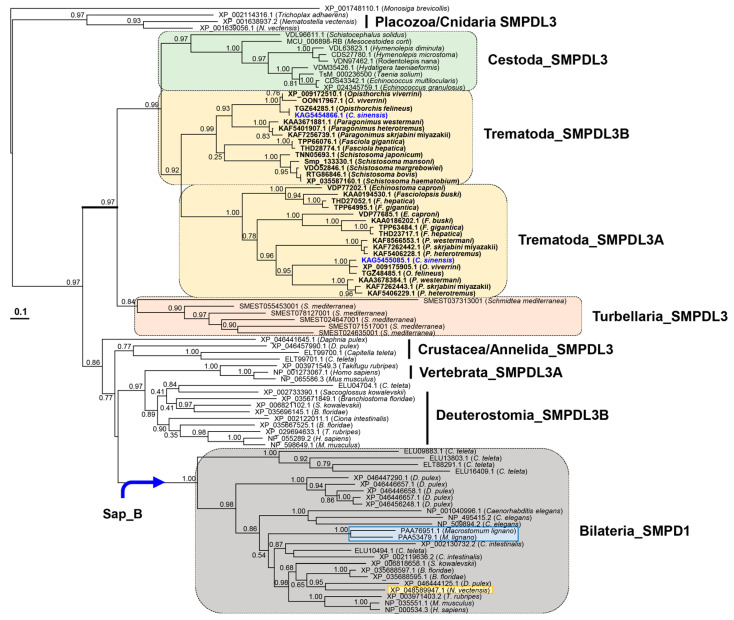
Phylogenetic analysis of SMPDL3 homologs across metazoans. The phylogenetic tree was constructed from amino acid sequences spanning the metallo-dependent phosphatase to ASM_C domains. The tree was rooted using a choanoflagellate homolog (*Monosiga brevicollis*, XP_001748110.1). Bootstrap values from 1000 replicates are shown at internal nodes. The Platyhelminthes SMPDL3 homologs are highlighted with thickened branches, and colored boxes indicate taxa representing major classes of the phylum. *Clonorchis sinensis* proteins are shown in blue. Light blue and yellow boxes within the bilateria_SMPD1 clade indicate homologs from *Macrostomum lignano* and *Nematostella vectensis*, respectively.

A maximum-likelihood tree was constructed using non-redundant Cs_SMPDL3A homologs (Figure 6; Supplementary Figure S2). The phylogeny indicated that the underlying *SMPDL3* and *SMPD1* genes had diverged from an ancestral *SMPDL3*-like gene, following the evolutionary trajectories of their respective host lineages from basal protozoans to vertebrates. The ancestral *SMPDL3* gene began to duplicate at least in cnidarians such as *Nematostella vectensis*, and one of the duplicated gene copies diversified into *SMPD1* gene lineage by acquiring an exonic segment encoding the Sap_B domain (blue arrow in Figure 6). Notably, the copy number of *SMPDL3* homologs differed among the three major platyhelminth classes. The free-living turbellarian *Schmidtea mediterranea* contained four paralogs, whereas parasitic trematodes and cestodes possessed two (*SMPDL3A* and *SMPDL3B*) and one *SMPDL3*-like gene, respectively. None of these flatworms retained the *SMPD1* gene. In contrast, another turbellarian, *Macrostomum lignano*, lacked *SMPDL3* but harbored two *SMPD1* copies (light-blue box). Together with the tight clustering of platyhelminth proteins apart from bilaterian orthologs, these observations suggested that *SMPDL3* genes in flatworms had undergone lineage-specific diversification independent of that in deuterostomians. Interestingly, the deuterostomian *SMPDL3A* was detected only in vertebrates, further reflecting the mosaic pattern of gene duplication and loss across the superphylum Deuterstomia.

**Figure 7:**
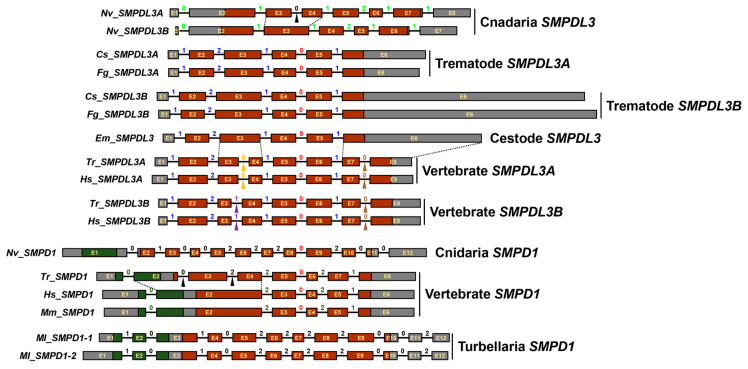
Exon–intron architectures of *SMPDL3* homologs. Exons (E) are shown as proportionally scaled rectangles according to nucleotide length, while introns are represented by lines of uniform length. Exonic regions encoding the Sap_B and metallo-dependent phosphatase domains are indicated in green and rust red, respectively. Intron phases are displayed above introns, with color patterns reflecting orthologous relationships, while black numerals indicate species-specific introns. Species- or lineage-specific intron insertions are marked by colored arrowheads.

Despite these multifaceted evolutionary trajectories, the exon-intron architectures were well conserved among the platyhelminth and vertebrate *SMPDL3* orthologs. All platyhelminth introns were orthologous to those of vertebrate genes in both position and phase (blue and red in Figure 7), although additional introns, either shared (brown arrowheads) or unique (orange and purple arrowhead), were observed in the vertebrate *SMPDL3A* and/or *SMPDL3B* genes. Meanwhile, the vertebrate *SMPDL1* genes contained introns that were non-orthologous to those of *SMPDL3*, except for a single shared intron (red). The *N. vectensis* and *M. lignano* genes also harbored unique introns not found in the platyhelminth and vertebrate counterparts (Figure 7).

### 2.7. Expression Profiles of the SMPDL3-like Genes in C. Sinensis and Human Cells

**Figure 8:**
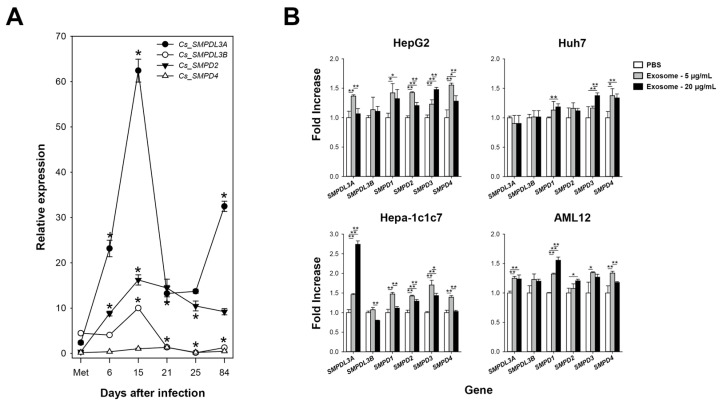
Expression profiles of *SMPDL3* and *SMPD* homologs in *Clonorchis sinensis* and hepatocyte cell lines. (**A**) Temporal transcript levels of *Cs_SMPDL3A*, *Cs_SMPDL3B*, *Cs_SMPD2*, and *Cs_SMPD4* across developmental and maturation stages, from metacercariae to 84-day adults. Asterisks indicate statistical significance (* *p* < 0.01). (**B**) Fold induction of *SMPDL3* and *SMPD* genes in human (HepG2, Huh7) and mouse (Hepa-1c1c7, AML12) hepatocyte cell lines treated with *C. sinensis* exosomes (5 or 20 μg/mL for 8 h). Asterisks denote statistical significance (* *p* < 0.05; ** *p* < 0.01).

Temporal expression profiles of *Cs_SMPDL3A* and *Cs_SMPDL3B* were examined in *C. sinensis* worms at various developmental and maturation stages, together with those of neutral sphingomyelinases *CS_SMPD2* (protein id. KAG5444124.1) and *Cs_SMPD4* (KAG5450648.1) identified in the *C. sinensis* proteome. The relative transcript levels of *Cs_SMPDL3A* and *Cs_SMPDL3B* markedly increased from the metacercarial stage (2.4 and 4.5, respectively) to the 15-day-old worm (62.4 and 10.0), and then decreased in the 21-day (13.2 and 1.4) and 25-day (13.7 and 0.2) worms. However, the expression levels were significantly re-elevated to 32.5 and 1.3, respectively, in the 84-day worm compared with the 25-day worm. *Cs_SMPD2* and *Cs_SMPD4* exhibited ontogenic profiles similar to those of *Cs_SMPDL3A* and *Cs_SMPDL3B*, although *Cs_SMPD2* maintained a low expression level throughout the later adulthood stages. Moreover, *Cs_SMPD4* expression remained consistently low (0.2–1.3) across all developmental stages examined (Figure 8A).

The effects of *C. sinensis* exosomes on *SMPDL3* homolog expression were assessed in hepatocyte-derived cell lines from human (HepG2 and Huh7) and mouse (Hepa-1c1c7 and AML12). In these cells, the transcription of *SMPDL3A* and other *SMPD* genes was slightly but significantly upregulated in response to the exosome treatment (*p* < 0.05), while the degree of induction varied among genes and cell types. Notably, higher concentration of the parasitic exosomes suppressed the expression of these genes. In contrast, *SMPDL3B* expression was minimally affected by the exosomal stimuli (Figure 8B). Similar induction patterns were observed in repeated experiemnts using HepG2 and Hepa-1c1c7 cells.

## 3. Discussion

Excretory-secretory products of animal cells contain heterogenous populations of EVs. Among these vesicles, microvesicles (50–1000 nm) are generated by direct outward budding of the plasma membrane and participate in coagulation, inflammation, and molecular transfer but lack definitive molecular markers [29]. In contrast, exosomes (30–100 nm) originate from the fusion of multivesicular bodies with the plasma membrane and typically harbor specific proteins such as CD9, CD63, and Tsg101 [30]. Exosomes actively facilitate immune modulation and macromolecule exchange through fusion with target cells, enabling the delivery of functional cargo [17]. In this study, an aqueous precipitation-based method was used to isolate exosomes from *C. sinensis* ESPs [31,32], following a high-speed pre-clearing centrifugation step to remove larger cellular debris and large EVs. The recovered vesicles exhibited a size range of 40–90 nm and contained exosome-specific proteins including CD63 (OG081 in Supplementary Table S6) and heat-shock protein (OG038), supporting enrichment of small EVs with exosomal characteristics. Nevertheless, it is recognized that precipitation-based isolation does not yield exclusively pure exosomes, and that residual contamination by other small EV subtypes as well as highly abundant soluble ESP proteins may persist. In particular, myoglobin, which constitutes a major proportion of total ESP proteins, could not be completely eliminated despite repeated precipitation steps. In this study, the term “exosomes” is used operationally to refer to an exosome-enriched EV fraction, because the isolated vesicles fall within the canonical exosomal size range and exhibit representative exosome-associated marker proteins, despite the inherent limitations of precipitation-based isolation.

The *C. sinensis* genomes were independently assembled for Korean (PRJNA386618, [33]) and Chinese (PRJDA72781, [34]) isolates. Their respective assemblies (GCA_003604175.2 and GCA_000236345.1) yielded comparable numbers of predicted genes (13,489 vs. 13,634), despite differences in sequencing depth, scaffolding, and assembly strategies (Hi-C/Nanopore with 3D-DNA vs. Illumina with Celera Assembler). However, the two proteomes exhibited distinct resolving power in homology-based searches such as BLAST (ver. 2.17.0+) against mRNA-derived sequences. For instance, a 100-aa fragment corresponding to residues 51–150 of CsMb1 [24] was found only in the Chinese proteome (GAA56719.1), whereas CsGST-σ3 [23] appeared exclusively in the Korean dataset (KAG5453121.1). Gene pools in both datasets were constructed using similar strategies involving ab initio prediction followed by verification based on transcriptome data [33,35]. Therefore, the difference in prediction depth is likely attributable to the sequencing depth and coverage of the respective transcriptomes, rather than genuine sequence divergence. To enhance identification reliability, both proteomes were therefore used for exosomal protein discovery, although the *C. sinensis* worms analyzed in this study originated from an endemic Korean area identical to that of the Korean genome project. Most proteins were shared between the two datasets, but a minor subset was unique to one (Supplementary Tables S4 and S5), reflecting differences in annotation depth and exon-intron prediction accuracy, as previously noted in flatworm genomes with compact exons and extensive repetitive elements [8,36]. Reciprocal BLAST validation and orthogroup reconciliation largely resolved these discrepancies, yielding a refined, nonredundant list of exosomal proteins (Supplementary Table S6) that improved confidence in subsequent functional and comparative analyses. Functional categorization of these proteins was performed using GO slim mapping to facilitate high-level comparisons across datasets. Although the yeast GO slim was selected because it provided substantially broader coverage than the generic slim, it represents a coarse-grained functional abstraction and was not intended to capture parasite- or host-specific biological nuances. Accordingly, GO-based summaries in this study are interpreted as descriptive indicators of functional trends rather than definitive pathway assignments; thus, individual protein functions need to be further evaluated in the context of known parasite biology and published functional evidence in future investigations.

Despite numerous unique proteins, an earlier *C. sinensis* exosomal proteome from Chinese isolates [20] shows substantial similarity to the present dataset from Korean isolates, converging on conserved exosomal functions related to cytoskeletal remodeling, metabolism, and host–parasite interaction. This concordance suggests that methodological differences mainly affected detection sensitivity rather than biological interpretation. Collectively, current evidence indicates that *C. sinensis* exosomes function as multifactorial mediators combining protein, lipid, and RNA cargoes to regulate biliary inflammation and tissue remodeling. Similar to the activation of epithelial cells by exosomes via the TLR9–ERK signaling pathway [19] and the induction of macrophage M1 polarization by vesicle-borne microRNA Csi-let-7a-5p [37], exosomal proteins such as heat-shock proteins (HSPs) and annexins may trigger receptor-mediated phosphorylation cascades upstream of host 14-3-3 scaffolds to generate downstream MAPK/ERK signaling. Glycolytic and cytoskeletal proteins such as enolase, actin, and tubulin likely support vesicle formation and energy-dependent signaling [38], while proteases and ubiquitin-related enzymes may influence antigen processing and presentation by modifying endosomal proteolytic or ubiquitin-dependent signaling [39,40,41]. The overlap between these functional observations and our GO enrichment for proteolysis, microtubule-based processes, and protein binding supports a view of the *Clonorchis* exosomes as dynamic molecular interfaces modulating both host–cell signaling and parasite metabolism. Similar proteomic profiles have been reported in *O. viverrini* [21], and *F. gigantica* [22], reflecting a conserved framework for vesicle formation, nutrient exchange, and immune communication that underpins the adaptive survival of these hepatobiliary trematodes within the bile-duct microenvironment [41,42,43]. In this context, HSP, annexins, and cathepsin-like proteases identified in this study (Supplementary Table S6) represent strong candidates for future investigations into the molecular mechanisms of host–cell activation, vesicle biogenesis, and immune modulation mediated by *C. sinensis* exosomes.

Sphingolipids are a class of lipids containing sphingoid backbone that provide the outer leaflet of plasma membranes with mechanical stability and chemical resistance in eukaryotic cells. Certain sphingolipid derivatives such as ceramide and sphingosine-1-phosphate are intimately involved in the cellular recognition and signaling processes that regulate cell proliferation, stress responses, and vesicle dynamics [44,45]. These molecules have also been detected in parasitic trematodes [46] and cestodes [47]. However, recent studies propose that the anabolic pathways of sphingolipids are highly dependent on host-derived precursors, likely reflecting the high energetic cost of de novo synthesis, similar to that of fatty-acid biosynthesis [10]. Indeed, genomic analyses of *C. sinensis* reveal incomplete repertoires of core biosynthetic proteins such as serine palmitoyltransferase and sphingomyelin (SM) synthase, whereas catabolic enzymes such as SMPD2/4 and SMPDL3 homologs persist [11]. These genetic losses suggest that *C. sinensis* and related trematodes have largely abandoned energy-intensive anabolic routes and instead rely on salvage and remodeling pathways that recycle host-derived SM and/or ceramide. The worms likely achieve this by secreting hydrolases such as sphingomyelinases or lipases, or by releasing factors that activate host enzymes, while lipid-binding transporters like FABPs may facilitate the uptake of liberated hydrophobic catabolites across the tegument [10,48]. These metabolites may sustain parasitic membrane biogenesis and exosomal lipid homeostasis, while also acting on host epithelial and immune cells to promote inflammatory and fibrotic responses through NF-κB, MAPK/ERK, and TGF-β/Smad signaling pathways [49,50]. Thus, the parasite’s lipid-scavenging strategy simultaneously fulfills its metabolic requirements and contributes to the characteristic biliary inflammation and fibrotic remodeling observed during clonorchiasis.

Relatively low but tegument-enriched distribution of sphingolipids in parasitic flatworms [51,52,53,54] support the long-standing view that trematodes acquire SM or its derivatives from surrounding host environments through the teguments [55] for assimilation into membrane sphingolipids. Nevertheless, the molecular machinery underlying these processes remains largely elusive. Genes for SMPD1 and ectonucleotide pyrophosphatase/phosphodiesterase 7 (ENPP7, also known as alkaline sphingomyelinase) homologs, key enzymes catalyzing the initial cleavage of dietary SM in the lysosome and intestinal lumen [56,57], respectively, as well as SM synthase are not identified in the *C. sinensis* genomes [11], suggesting the presence of alternative mechanisms supporting sphingolipid metabolism. Instead, the worm possesses genes for SMPDL3A/3B and SM synthase-related protein 1 (SMSr1, KAG5447477.1).

SMPDL3A tightly conserves core structural features including the di-zinc catalytic center characterized in human SMPDL3A and SMPD1 (Figure 4 and Figure 5), even though the SMPDL3 homologs appear to have evolved through polytomic, rather than phyletic, routes across animal phyla (Figure 6 and Figure 7). This pattern implies repeated lineage-specific functional diversifications, possibly driven by distinct nutritional environments and following adaptation in platyhelminths. Unlike SMPD1, however, the SMPDL3A proteins have been known to exhibit nucleotide hydrolysis activity [58], mainly due to the absence of the saponin B-type domain and Pro-rich linker (Figure 4 and Figure 5) that enclose the catalytic cleft to form a hydrophobic SM-binding pocket in SMPD1 [27,28]. In contrast, Cs_SMPDL3A contains a hydrophobic domain, termed TM_EGFR-like helix [26], in the C-terminus (Figure 4). The domain likely anchors the enzyme to the exosomal or tegumental membrane, positioning its catalytic site at the lipid-water interface. In this configuration, the adjacent bilayer could transiently create a hydrophobic microenvironment permitting limited access of SM head groups to the di-zinc center, as reported for membrane-associated phospholipases [59]. Alternatively, exosomal Cs_SMPDL3A or associated factors may enhance host SMPD2–4 activity, as evidenced by this study (Figure 8B), generating membrane SM-derived ceramides. If the Cs_SMSr1 protein also exhibits weak activity for assimilating ceramide into SM or its analogs such as ceramide phosphoethanolamine, a sphingolipid species often more abundant than SM in many invertebrates [60,61], then Cs_SMPDL3A and Cs_SMSr1 together could sustain low-level sphingolipid turnover consistent with the limited sphingolipid content in *C. sinensis* [51]. The resulting ceramide production in a host–parasite interface may modulate host–cell signaling pathways involved in inflammation and tissue remodeling, thereby contributing to the parasite’s adaptation within the biliary niche. Further biochemical characterization of these enzymes will clarify their catalytic mechanisms and physiological roles in *C. sinensis*.

## 4. Materials and Methods

### 4.1. Preparations of C. sinensis Excretory–Secretory Products and Exosomal Fraction

Live *C. sinensis* worms (>300 worms), obtained from experimentally infected rats at 5 weeks post-infection as described previously [62], were washed five times with ice-cold physiological saline and incubated in 10 mL RPMI-1640 medium (phenol red- and serum-free, pH 7.2; Thermo Fisher Scientific, Waltham, MA, USA) containing antibiotics (100 U/mL penicillin, and 100 µg/mL streptomycin), at 37 °C in a 5% CO_2_ incubator. After 1 h incubation, the medium was replaced with fresh identical medium and further incubated for 4 h. The worm-conditioned medium was centrifuged at 2000 rpm for 10 min to remove eggs, sperms, and other cellular debris, and subsequently at 12,000 rpm for 40 min at 4 °C. Resulting supernatant containing the *C*. *sinensis* ESP was used directly in the isolation of exosomes. The ESP solution was mixed with the Total Exosome Isolation Reagent (Invitrogen, Gaithersburg, MD, USA) and incubated overnight at 4 °C. The mixture was centrifuged at 12,000 rpm for 1 h at 4 °C. The precipitated pellet was resuspended in PBS and subjected to a second precipitation step using the same reagent. After incubation for 20 min at room temperature, the pellet was collected by centrifugation and resuspended in pre-cold PBS. The resulting exosome-enriched solution, referred to as the exosomal fraction in this study, was stored at −80 °C until use.

### 4.2. Transmission Electron Microscopy

The freshly isolated exosomes were put on a copper grid coated with 0.125% formvar in chloroform and placed at room temperature to dry for fixation. The grids were stained with 1% *v*/*v* uranyl acetate in double distilled water and observed under the JEM 1011 transmission electron microscope (JEOL USA, Peabody, MA, USA) at 75 kV.

### 4.3. One-Dimensional SDS-PAGE and Western Blotting

Proteins in whole ESP (10 μg/lane) and exosomal fraction (1.5 and 10 μg/lane) were separated on 10–15% gels by one-dimensional SDS-PAGE under reducing conditions. Protein concentrations were determined using the Lowry method with bovine serum albumin as a standard, and all measurements were performed within the linear range of the assay. After electrophoresis, proteins were visualized by staining the gels with the ProteoSilver Silver Stain Kit (Sigma-Aldrich, St. Louis, MO, USA) according to the manufacturer’s instructions, or transferred onto nitrocellulose membranes (Schleicher & Schuell Bioscience, Dassel, Germany). The protein blots were reacted with a mixture of mouse antisera specific to *C. sinensis* myoglobin (CsMb1, [24]) and sigma-class glutathione transferase (CsGST-σ3, [23]), and then with horseradish peroxidase-conjugated rabbit anti-mouse IgG antibody (Bethyl Laboratories, Montgomery, TX, USA). Positive signals were developed with an enhanced chemiluminescence detection reagents (GE Healthcare Life Science, Pittsburgh, PA, USA) and visualized using an Amersham ImageQuant 800 imaging system (Cytiva, Marlborough, MA, USA). Whole ESP and exosomal fraction treated with trypsin (1 mg/mL) for 10, 20, or 40 min at 37 °C were also applied in the SDS-PAGE and subsequent Western blot analysis.

### 4.4. In-Gel Digestion with Trypsin and Extraction of Peptides

Protein bands were manually excised from the silver nitrate-stained gel and processed for in-gel trypsin digestion following established protocols [63]. Excised gel fragments were washed for 1 h at room temperature in 25 mM ammonium bicarbonate buffer (pH 7.8) containing 50% (*v*/*v*) acetonitrile (ACN), then dehydrated for 10 min in a centrifugal vacuum concentrator (Biotron, Incheon, Republic of Korea). The dried fragments were rehydrated in 50 ng of sequencing-grade trypsin solution (Promega, Madison, WI, USA) and incubated overnight at 37 °C in 25 mM ammonium bicarbonate buffer (pH 7.8). Peptides generated by the digestion were extracted for 20 min with 100 μL of 1% formic acid (FA) with 50% ACN under mild sonication, and the extracts were concentrated by vacuum centrifugation. Prior to LC-MS/MS analysis, samples were desalted on a reversed-phase microcolumn as described by Gobom et al. [64]. The column was pre-equilibrated with 10 μL of 5% FA, washed with the same solution after peptide loading, and peptides were finally eluted in 8 μL of 70% ACN containing 5% FA.

### 4.5. Identification of Proteins by LC-MS/MS

Peptide mixtures were analyzed on a nanoACQUITY UPLC system coupled to an LTQ-Orbitrap mass spectrometer (Thermo Fisher Scientific, Bremen, Germany). Chromatographic separation was carried out on a BEH C18 column (1.7 μm, 100 μm × 100 mm; Waters, Milford, MA, USA) with a binary solvent system consisting of 0.1% FA in water (solvent A) and 0.1% FA in acetonitrile (solvent B). The gradient was programmed as 10–40% B over 21 min, 40–95% B over 7 min, and re-equilibration to 10% B for 10 min, at a flow rate of 0.5 μL/min. Mass spectra were acquired in a data-dependent mode, with MS scans (*m*/*z* 300–2000) followed by MS/MS of selected ions. Each MS/MS spectrum represented the average of a single microscan. Instrument parameters included an ion transfer tube temperature of 275 °C, spray voltage of 2.3 kV, and normalized collision energy of 35%. The resulting spectra were processed with the SEQUEST algorithm [65] and searched against the *C. sinensis* protein database sets in GenBank (https://www.ncbi.nlm.nih.gov/), which were derived from two independent genome projects PRJNA386618 (Korean isolate) and PRJDA72781 (Chinese isolate), using the MASCOT search engine (Matrix Science, London, UK). Search settings specified carbamidomethylation (C) of cysteine as a fixed modification, and deamidation (N/Q) and oxidation (M) as variable modifications. Peptide mass tolerances were 10 ppm for precursor ions and 0.8 Da for MS/MS ions. One missed cleavage was allowed, and charge states of +2 and +3 were considered. Only peptide matches that met the MASCOT probability threshold were accepted.

### 4.6. Gene Ontology Annotation and Classification

Amino acid sequences of the *Clonorchis* proteins identified by LC-MS/MS were analyzed using InterProScan (ver. 5.73-104.0; [66]) to assign Gene Ontology (GO) terms across the three major categories: biological process, molecular function, and cellular component. The program outputted both specific (child) terms and their ancestral parent terms along the GO hierarchy, resulting in raw annotations with hierarchical redundancy. To classify these terms into higher-level functional categories and reduce redundancy, multiple GO slim subsets were tested. The generic slim (goslim_generic.obo) provided poor coverage (<10%), whereas the yeast slim (goslim_yeast.obo) yielded >95% mapping. Although originally designed for yeast, this slim encompasses broadly applicable functional categories across eukaryotes and was therefore selected to ensure comprehensive coverage. The most recent versions of the full ontology (go-basic.obo) and the yeast slim were retrieved from the Gene Ontology Consortium (https://www.geneontology.org/docs/download-ontology/, accessed on 1 September 2025). Using these resources, category- and subcategory-level distributions of the *Clonorchis* proteins were determined from the mapped GO terms. In addition, to highlight the most specific functional assignments, terminal GO terms (i.e., leaf nodes without downstream children in the ontology) were extracted from the InterProScan annotations. These terminal terms were grouped at an adaptive ontology depth to balance detail with interpretability, and the 15 most frequent categories were summarized to compare the functional repertoires between datasets.

### 4.7. Structural Characterization of SMPDL3A and Its Homologs

Amino acid sequences of *Clonorchis* SMPDL3 proteins were aligned with those of human homologs (Hs_SMPDL3A, NP_001273067.1; Hs_SMPDL3B, NP_055289.2; and Hs_SMPD1, NP_000534.3) using the MUSCLE program (https://www.ebi.ac.uk/jdispatcher/msa/muscle, accessed on 16 September 2025). Functional motifs and conserved signatures were identified in the alignment with reference to previous studies on human SMPDL3A and SMPD1 [27,28]. Prediction of transmembrane regions and hydrophobic leader peptides was performed using the DeepTMHMM server (ver. 1.0; https://services.healthtech.dtu.dk/services/DeepTMHMM-1.0/, accessed on 16 September 2025). The tertiary structures of the *Clonorchis* proteins were modeled using AlphaFold (https://alphafoldserver.com/; [67]). Among multiple structural models generated by the program, the most reliable one was selected based on a comparative assessment of the predicted global confidence (pTM) and per-residue predicted Local Distance Difference Test (pLDDT) scores. The stereochemical quality of the selected models was validated using PROCHECK (ver. v.3.5.4; https://www.ebi.ac.uk/thornton-srv/databases/pdbsum/Generate.html, accessed on 24 September 2025), and their overall conformations were compared with the crystal structures of Hs_SMPDL3A (PDB ID: 5EBB) and Hs_SMPD1 (5I81) available in the RCSB Protein Data Bank (https://www.rcsb.org/) using UCSF ChimeraX [68]. Structural similarity was evaluated by calculating the root-mean-square deviation (RMSD) between Cα-atom positions. Both pruned RMSD values, calculated after outlier exclusion, and RMSD values across all aligned residues were obtained.

### 4.8. Phylogenetic and Genomic Interpretation

Cs_SMPDL3A homologs were identified in GenBank proteomes representing major animal phyla, including multiple platyhelminth species (Supplementary Figure S2), through BLASTp searches (ver. 2.17.0+). For *S. mediterranea*, homologous mRNA sequences retrieved from the WormBase Parasite transcriptomes (ver. WS291; https://parasite.wormbase.org/index.html, accessed on 15 August 2025) by tBLASTn searches were translated into putative proteins. The aa sequences were aligned using MUSCLE, and a conserved region encompassing the metallo-dependent phosphatase to ASM_C domains, which had been annotated by InterProScan, was extracted for phylogenetic analysis. Phylogenetic inference was performed using PhyML (ver. 3.1, [69]) under the Jones-Taylor-Thornton (JTT) model, incorporating a gamma-distributed rate variation (G) and empirical aa frequencies (F). Sites with excessive gaps were partially excluded (site coverage cutoff = 90%), and branch support was estimated by 1000-replicate bootstrap analysis. The resulting tree was visualized with TreeView [70].

Exon-intron organizations of *SMPDL3* homologs were reconstructed by aligning their genomic and transcript sequences. Splice junctions were verified according to the canonical GT-AG rule [71]. Intron insertion phases relative to codon boundaries were classified as phase 0 (between codons), phase 1 (between the first and second nucleotides of a codon), or phase 2 (between the second and third nucleotides). The positions and phases of introns were then mapped onto the aligned aa sequences to assess orthologous relationships and structural conservation across taxa.

### 4.9. Quantitative PCR Analysis of Gene Expression

Total RNAs were extracted from *C. sinensis* worms at various developmental stages (>30 worms per stage) using QIAzol reagent and the RNeasy Mini Kit (Qiagen, Hilden, Germany), as described previously [24], and treated with an RNase-free DNase (New England Biolabs, Ipswich, MA, USA). cDNAs were synthesized with the iScript cDNA Synthesis Kit (1 μg of total RNA per 10 μL reaction; Bio-Rad, Munich, Germany). Relative expression levels of *SMPDL3A*, *SMPDL3B*, *SMPD2*, and *SMPD4* were determined by qPCR using gene-specific primers (Supplementary Table S7). Primers were designed to span exon-exon junctions to avoid amplification of contaminating genomic DNA. The β-actin gene (protein id. KAG5449685.1) served as an internal reference. The amount of cDNA used as template (i.e., dilution rate) was empirically optimized based on the target gene exhibiting the lowest expression level, such that its Cq value remained below 30. This optimized template concentration was applied uniformly across all target genes to allow direct comparison of relative expression levels. PCR reactions were performed in triplicate with SYBR Green Master Mix on a CFX96 system (Bio-Rad, Hercules, CA, USA). Melt-curve analysis confirmed single amplicons. Relative expression was calculated by the 2^−ΔΔCt^ method [72], and data are presented as mean ± SD. Statistical significance was evaluated by two-tailed Student’s *t*-test (*p* < 0.05).

Human (HepG2, Huh7) and mouse (Hepa-1c1c7, AML12) hepatocyte cell lines were seeded in 12-well culture plates (1 × 10^5^ cells/well) in appropriate media: DMEM for HepG2 and Huh7, α-MEM for Hepa-1c1c7, or DMEM/F-12 for AML12, each supplemented with 10% fetal bovine serum, 100 U/mL penicillin, and 100 µg/mL streptomycin (Thermo Fisher Scientific). Medium for AML12 additionally included insulin (5 µg/mL), transferrin (5 µg/mL), selenium (5 ng/mL), and dexamethasone (40 ng/mL) (Thermo Fisher Scientific). Cells were incubated at 37 °C in 5% CO_2_ and treated with *C. sinensis* exosomes (5 or 20 µg/mL) for 8 h upon reaching approximately 80% confluence. For each treatment, three independent culture replicates were prepared and pooled prior to RNA extraction to minimize preparation-related variability. The pooled RNAs samples were then subjected to the qPCR analysis of *SMPDL3* and *SMPD* transcripts using primer sets listed in Supplementary Table S7, as described above. The measurements were performed twice using independently cultured HepG2 and Hepa-1c1c7 cells.

## 5. Conclusions

This study provides an integrated characterization of exosomes derived from *C. sinensis* Korean isolates, demonstrating conserved vesicle morphology, marker expression, and protein compositions enriched for cytoskeletal, metabolic, and vesicle-trafficking functions associated with epithelial signaling and host–parasite communication. Among these components, SMPDL3A emerged as a key candidate enzyme potentially involved in sphingolipid turnover in the bile ducts infected with SMPD1-lacking *C. sinensis*, proposed based on structural comparisons and the lineage-specific evolutionary trajectories of SMPDL3 homologs including SMPDL3A, SMPDL3B, and SMPD1. Although the tightly conserved catalytic architecture and C-terminal transmembrane segment likely support its potential role as a sphingomyelinase, enzymatic verification was not achieved in this study because its recombinant form could not be produced in *Escherichia coli* or *Pichia pastoris*. Transcriptomic analyses of *Cs_SMPDL3A*- and *Cs_SMPD2*-transfected cholangiocyte cells are currently underway, and forthcoming results are expected to elucidate their downstream molecular effects. Collectively, these findings advance understanding of *C. sinensis* exosome biology and highlight the potential contribution of exosomal enzymes to sphingolipid remodeling and host–cell responses during clonorchiasis.

## Data Availability

The data used to support the findings of this study are available from the corresponding author (Bae, Y.-A.) upon request.

## Supplementary Materials

The supporting information can be downloaded at: https://www.mdpi.com/article/10.3390/ijms27020682/s1.

## Abbreviations

The following abbreviations are used in this manuscript:

ASM	Acid sphingomyelinase
BP	Biological process
CC	Cellular component
EMT	Epithelial-mesenchymal transition
ENPP7	Ectonucleotide pyrophosphatase/phosphodiesterase 7
ESP	Excretory-secretory product
EV	Extracellular vesicle
GO	Gene ontology
LC-MS/MS	Liquid chromatography-tandem mass spectrometry
MF	Molecular function
PMF	Peptide mass fingerprinting
SM	Sphingomyelin
SMPD	Sphingomyelin phosphodiesterase
SMPDL3	Sphingomyelin phosphodiesterase acid-like 3
SMSr1	Sphingomyelin synthase-related protein 1
TEM	Transmission electron microscope
TM_EGFR	Transmembrane_epidermal growth factor receptor
