# Supplementary material for: Proteomic Profiling of an Exosome-Enriched Extracellular Vesicle Fraction and Structural Characterization of SMPDL3A in the Carcinogenic Liver Fluke Clonorchis sinensis
Source: Int J Mol Sci. 2026 Jan 9;27(2):682. doi: 10.3390/ijms27020682 (PMC12841337; doi:10.3390/ijms27020682)
Supplement: Supplementary file 1 [file ijms-27-00682-s001.zip › Suppl Figures.pptx]

## Slide 1
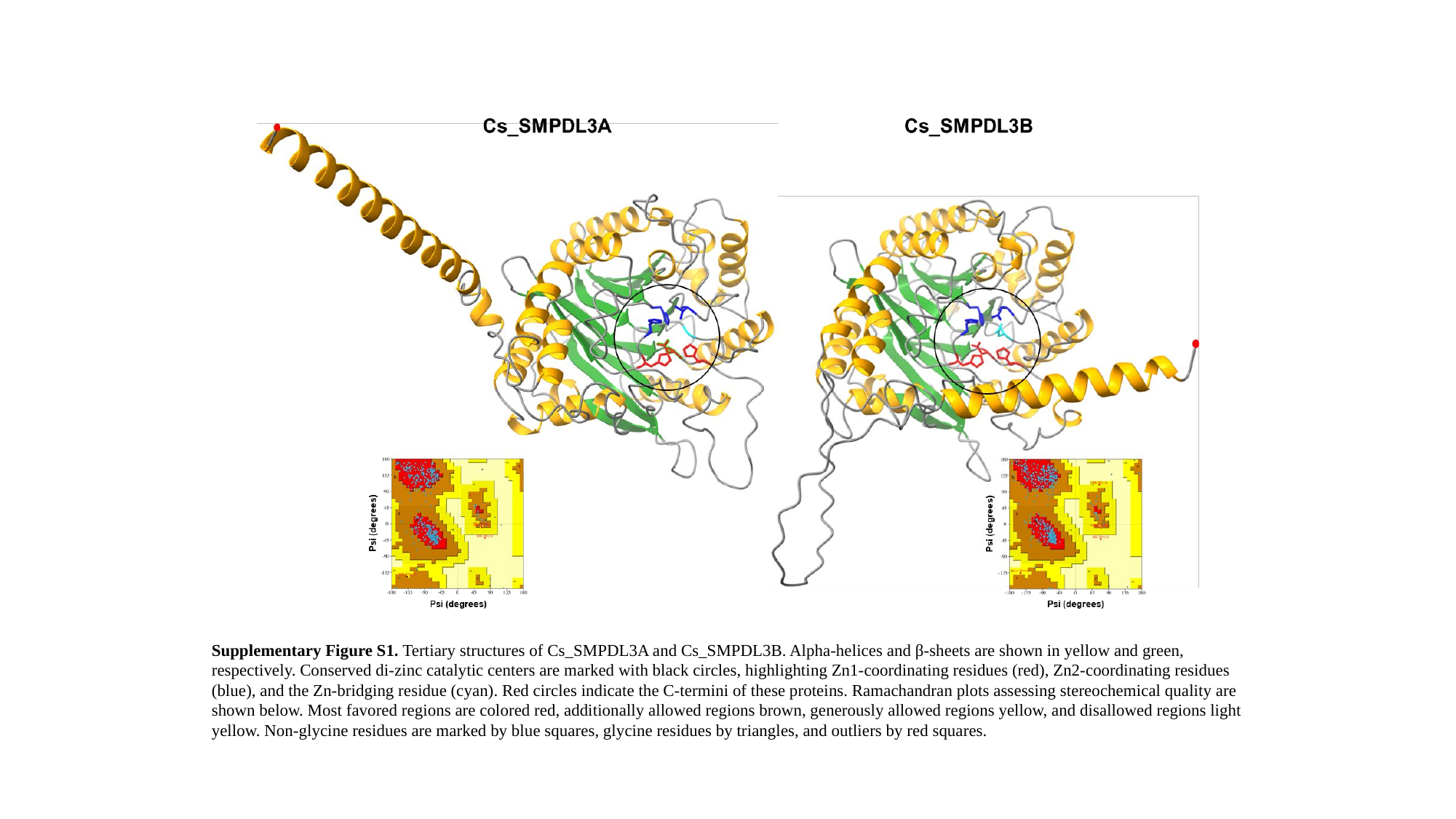

Supplementary Figure S1. Tertiary structures of Cs_SMPDL3A and Cs_SMPDL3B. Alpha-helices and β-sheets are shown in yellow and green, respectively. Conserved di-zinc catalytic centers are marked with black circles, highlighting Zn1-coordinating residues (red), Zn2-coordinating residues (blue), and the Zn-bridging residue (cyan). Red circles indicate the C-termini of these proteins. Ramachandran plots assessing stereochemical quality are shown below. Most favored regions are colored red, additionally allowed regions brown, generously allowed regions yellow, and disallowed regions light yellow. Non-glycine residues are marked by blue squares, glycine residues by triangles, and outliers by red squares.

## Slide 2
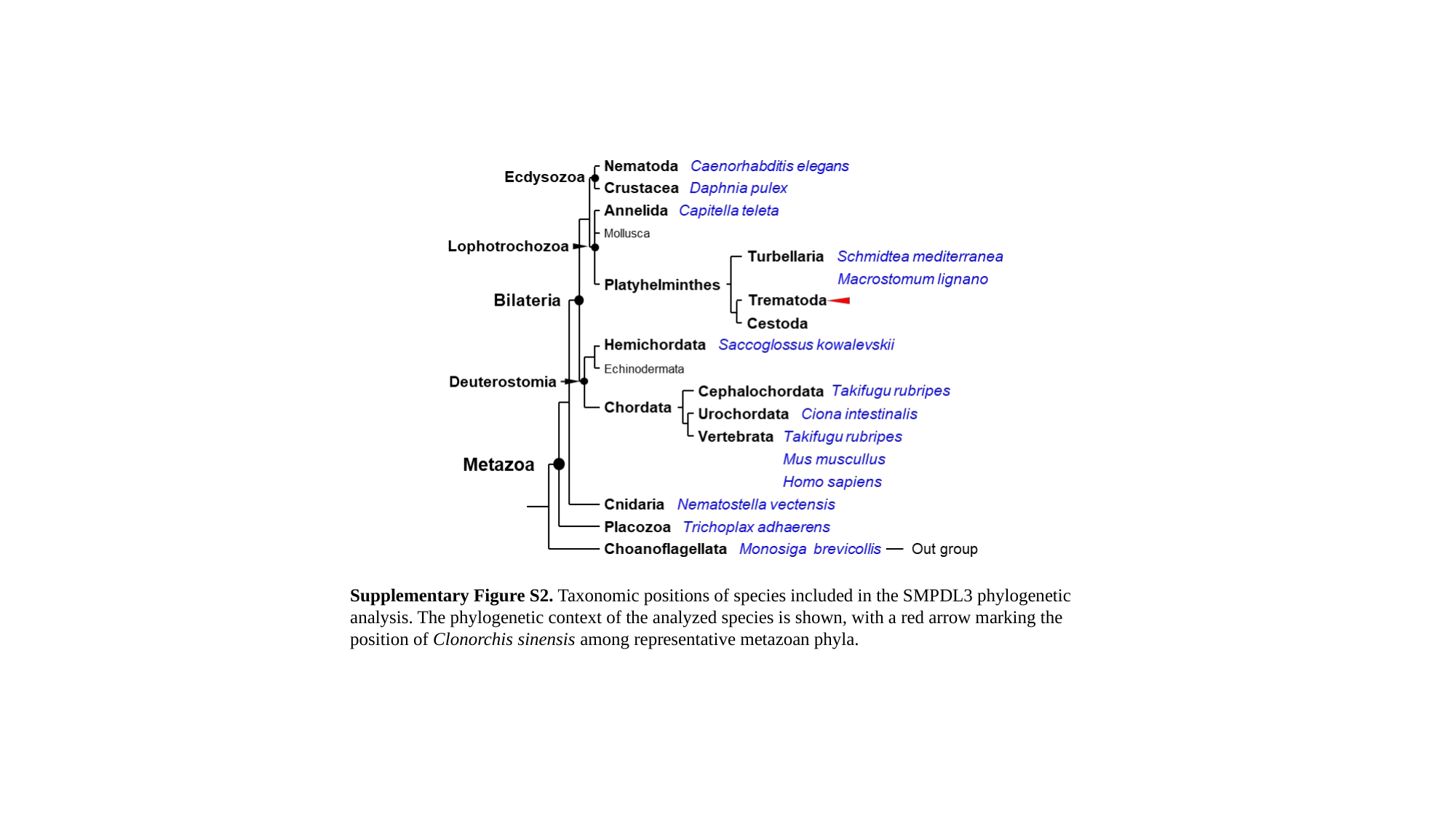

Supplementary Figure S2. Taxonomic positions of species included in the SMPDL3 phylogenetic analysis. The phylogenetic context of the analyzed species is shown, with a red arrow marking the position of Clonorchis sinensis among representative metazoan phyla.
